# Integration of Molecular Profiling and Chemical Imaging to Elucidate Fibroblast-Microenvironment Impact on Cancer Cell Phenotype and Endocrine Resistance in Breast Cancer

**DOI:** 10.1371/journal.pone.0096878

**Published:** 2014-05-09

**Authors:** Sarah E. Holton, Anna Bergamaschi, Benita S. Katzenellenbogen, Rohit Bhargava

**Affiliations:** 1 Department of Bioengineering, University of Illinois at Urbana-Champaign, Urbana, Illinois, United States of America; 2 Beckman Institute for Advanced Science and Technology, University of Illinois at Urbana-Champaign, Urbana, Illinois, United States of America; 3 Departments of Molecular and Integrative Physiology, Cell and Developmental Biology, University of Illinois at Urbana-Champaign, Urbana, Illinois, United States of America; 4 University of Illinois Cancer Center, University of Illinois at Urbana-Champaign, Urbana, Illinois, United States of America; 5 Departments of Mechanical Science and Engineering, Electrical and Computer Engineering, and Chemical and Biomolecular Engineering, University of Illinois at Urbana-Champaign, Urbana, Illinois, United States of America; King Faisal Specialist Hospital & Research center, Saudi Arabia

## Abstract

The tumor microenvironment is known to play a key role in altering the properties and behavior of nearby cancer cells. Its influence on resistance to endocrine therapy and cancer relapse, however, is poorly understood. Here we investigate the interaction of mammary fibroblasts and estrogen receptor-positive breast cancer cells in three-dimensional culture models in order to characterize gene expression, cellular changes, and the secreted protein factors involved in the cellular cross-talk. We show that fibroblasts, which are the predominant cell type found in the stroma adjacent to the cancer cells in a tumor, induce an epithelial-to-mesenchymal transition in the cancer cells, leading to hormone-independent growth, a more invasive phenotype, and resistance to endocrine therapy. Here, we applied a label-free chemical imaging modality, Fourier transform infrared (FT-IR) spectroscopic imaging, to identify cells that had transitioned to hormone-independent growth. Both the molecular and chemical profiles identified here were translated from cell culture to patient samples: a secreted protein signature was used to stratify patient populations based on gene expression and FT-IR was used to characterize breast tumor patient biopsies. Our findings underscore the role of mammary fibroblasts in promoting aggressiveness and endocrine therapy resistance in ER-positive breast cancers and highlight the utility of FT-IR for the further characterization of breast cancer samples.

## Introduction

More than 70% of breast cancers diagnosed in the US are estrogen receptor positive (ER^+^) [1], [2]. ER^+^ tumors generally have more favorable prognoses compared to other subtypes and can be treated with targeted endocrine therapies such as tamoxifen [3]. Though many ER^+^ patients initially respond favorably to targeted therapy, up to 30% of treated cancers recur [3], [4]. For patients with recurrent disease, the five-year survival rate drops to 20%, with a median survival of 12–24 months [5]. Therefore, it would be advantageous to identify at the time of initial diagnosis the patients who will not respond to endocrine therapy in the long-term so that their care can be managed differently. The factors underlying recurrence arising from endocrine resistance are not fully understood, but it is increasingly appreciated that the microenvironment of the tumor cells can play a critical role in impacting the behavior of the cancer cells [6], [7].

To understand the molecular factors driving endocrine resistance and tumor recurrence, we utilized three-dimensional cell co-culture models and studied them using molecular profiling and chemical imaging. We hypothesized that normal fibroblasts serve at the frontline of heterotypic interactions experienced by cancer cells because they are the first cell type encountered by dysplastic epithelium. Further, fibroblasts are encountered in the microenvironment during every stage of disease progression. The microenvironment is emerging as a new target for cancer therapies [8]. It is now clear that three-dimensional (3D) cultures represent a more realistic model for tumors [9], [10], and excellent 3D tumor models have been proposed [11], [12]. However, 3D co-cultures to study heterotypic interactions are less widely used [13], [14]. Hence, we developed and employed a series of 3D co-culture systems to investigate the impact of fibroblasts on tumor cell phenotype and response to endocrine therapy. Fibroblasts are the most abundant cell type in the breast stroma and while they play a role in the endocrine regulation of normal breast differentiation, it is not well understood how they affect the response of breast cancer cells to targeted endocrine therapy. In order to characterize the influences of cancer cell-stromal interactions on therapeutic response, we profiled the conditioned medium of the co-culture and defined a molecular interaction signature (iSig). The iSig provides mechanistic insight into tumor progression and the dynamics of cancer cell behavior by identifying specific secreted proteins involved in cancer cell-stromal cross-talk. When we separated breast cancer patient microarray data based on iSig expression levels, we were able to predict patient outcome that was comparable to available gene expression profiling methods.

Although uncovering genomic and proteomic dynamics of tumor behavior are crucial for understanding the pathophysiology of cancer, imaging techniques remain a gold standard of determining diagnosis and prognosis in many solid tumors, including breast cancer. Here, we used Fourier Transform infrared (FT-IR) spectroscopic imaging [15] for rapid and label-free profiling of co-culture samples, integrating molecular information about cellular phenotypes with microscopy. As opposed to antibody-based imaging approaches such as immunohistochemistry (IHC) or immunofluorescence, emerging chemical imaging technologies use spectroscopy to provide functional and molecular information within cells and tissues without staining or the requirement for *a priori* knowledge of molecular transformations. By visualizing the chemistry inherent within a sample, cell activity changes and disease states *in vitro* [16], [17] and cell types within complex tissues [18]–[20] can be monitored. Although a number of studies have reported the development of high performance imaging, rapid data processing and classification algorithms, as well as applications to histologic analyses of tissues [21]–[24], few studies have related spectral data to underlying molecular transformations.

In this study, we have monitored changes in gene expression and cell phenotype associated with cancer cell-fibroblast interactions and identified chemical changes with FT-IR spectroscopy in a “bottom up” approach to identify specific cellular contributions of the microenvironment on cancer cell behavior and response to endocrine therapy. The first part of this study reports the step-wise, validated development of a 3D co-culture model that put molecular conclusions in a spatial context. Next, we focus on molecular transformations and describe a secreted protein interaction signature that effectively predicts clinical outcomes in patient tumor samples. Finally, we develop the chemical imaging signature of the underlying genomic states in endocrine-responsive breast cancer. Together, these highlight a pivotal role for mammary fibroblasts located in the tumor cell microenvironment in determining the phenotypic properties of ER^+^ breast cancer cells and their sensitivity or resistance to endocrine therapy. Further, we show how chemical imaging may be used to rapidly identify these cellular phenotypes in heterogeneous samples.

## Results

### 3D culture systems were engineered to examine cancer cell-mammary fibroblast interactions

**Figure 1:**
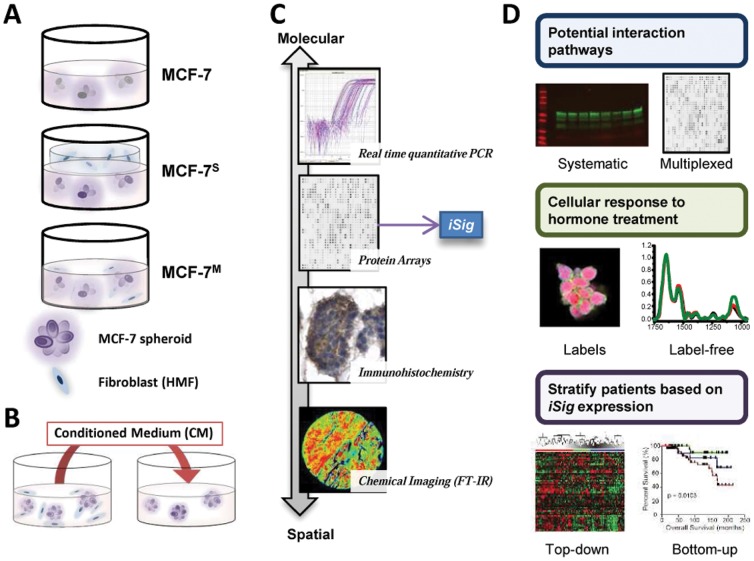
Three-dimensional co-culture models and analytical approaches in this study. Schematic of the several three-dimensional co-culture models we utilized to study the interactions between MCF-7 breast cancer cells and primary mammary fibroblasts. The MCF-7 were grown as spheroids in a Matrigel overlay culture. Fibroblasts were incorporated either in a direct-contact mixed culture (MCF-7^M^) or in a separate collagen layer in a sandwich culture (MCF-7^S^). To study the effects of paracrine signaling, conditioned medium (CM) studies were done in which CM was taken from the mixed culture and used to treat MCF-7 or normal mammary epithelial cells (HMEC) grown alone. The CM was also profiled using protein arrays to obtain the secreted protein interaction signature (iSig). We used gene expression and phenotypic assays to study response to hormone and the expression of markers of EMT. This molecular profiling approach was correlated to label-free FT-IR spectroscopic imaging and also gene expression from patient samples.

In order to study the interactions between ER^+^ breast cancer cells and mammary fibroblasts, we utilized four 3D culture models, shown schematically in Figure 1*A and 1B*. We used a number of approaches to translate these findings to human disease states (Fig. 1*C and 1D*). The breast cancer model was comprised of ER^+^ MCF-7 breast cancer cells grown on Matrigel to form 3D spheroid structures, denoted hereon simply as MCF-7. Primary human mammary fibroblasts (HMF), isolated from normal breast tissue, were commercially obtained. Several co-culture geometries were used in order to systematically characterize the mechanism by which tumor-adjacent stromal fibroblasts exert influence over cancer cell growth. An indirect co-culture, the ‘sandwich’ (MCF-7^S^), represents the compartmentalization of cancer cells and fibroblast-rich stroma, as may be observed in locally confined tumors. Because the fibroblasts are grown in a separate collagen layer, the two cell types are able to communicate continuously via molecular diffusion of soluble factors while remaining in their respective layers [16]. After a prescribed co-culture time, the layers are easily separated, and molecular analyses can be performed without any cell crossover. The direct, or ‘mixed’, co-culture (MCF-7^M^), consists of both MCF-7 and fibroblasts grown together on Matrigel, allowing for cell-cell interactions arising from direct contact. To understand the transient influence of soluble factors on cancer cell phenotypes, a conditioned medium (CM) culture was employed which consists of medium from MCF-7^M^ added to the MCF-7 cells. This medium was subsequently analyzed to identify the secreted factors that characterize cancer cell-fibroblast interactions in this model system. These models were used to comprehensively interrogate the differential fibroblast-cancer cell interactions that influence cancer cell behavior and resistance to therapy.

### Fibroblasts induce an epithelial-to-mesenchymal transition (EMT) in breast cancer cells

**Figure 2:**
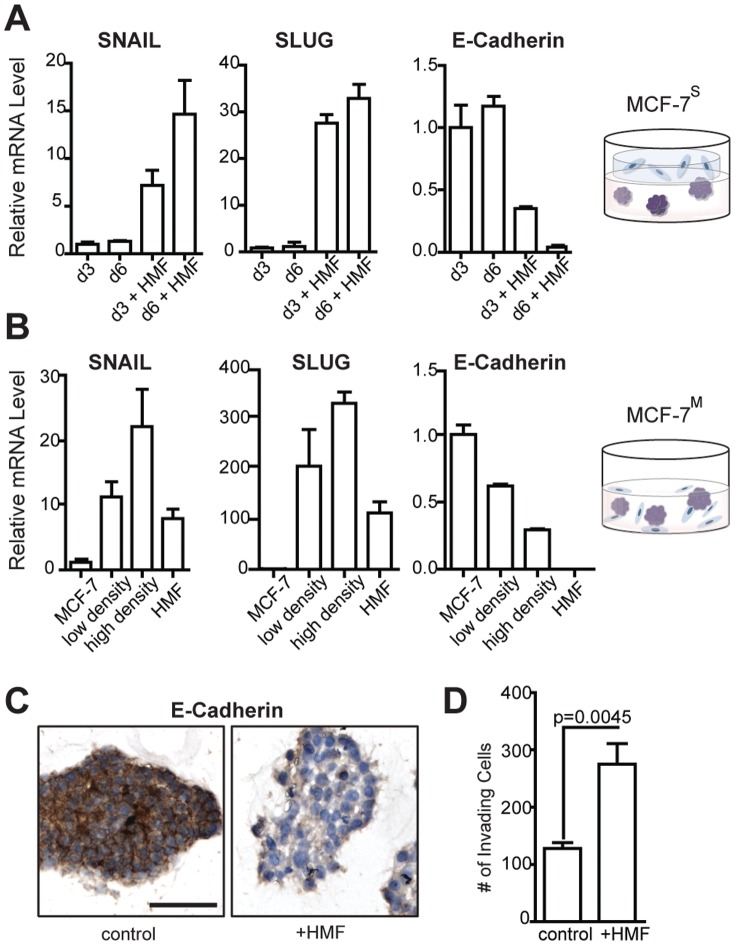
Mammary fibroblasts induce an epithelial-to-mesenchymal transition in ER^+^ breast cancer cells in 3D culture. (A) Over the course of 6 days in the sandwich co-culture, MCF-7^S^ display increased mRNA levels of EMT markers SNAIL and SLUG with a decrease in E-cadherin mRNA. (B) Mixed co-cultures were prepared at two fibroblast seeding densities, low- and high-density (relative to MCF-7) and EMT markers were modulated up or down as seen with the sandwich co-culture, and a dose-dependent response to the fibroblast presence was observed. (C) Immunohistochemistry was used to confirm decrease in overall E-cadherin protein level inMCF-7^M^ (D) Co-culture with human mammary fibroblasts (HMF) increases the invasiveness of MCF-7 breast cancer cells.

We observed that co-culture with HMF induced an epithelial-to-mesenchymal transition (EMT) in MCF-7 breast cancer cells. The cells grown in the MCF-7^S^ model displayed increased levels of SNAIL (7-fold) and SLUG (25-fold) mRNA as well as a 4-fold decrease of E-Cadherin mRNA (Fig. 2*A*), all characteristic of the mesenchymal cell phenotype after undergoing EMT [25]. The effects observed at 3 days were sustained at longer times, indicating that the induced EMT phenotype was not transient. This phenotype was accompanied by an increase in mRNA levels for growth factors, including amphiregulin (Figure S1 in File S1), which is often associated with a loss of ER and increased inflammatory signaling in breast cancers [26], [27].

To confirm that the phenotypic changes observed in MCF-7^S^ were due to fibroblast signaling and not dependent on the sandwich geometry, we also used the MCF-7^M^ culture. Similar to MCF-7 and MCF-7^S^, cells still grew as spheroids in the MCF-7^M^ model, with fibroblasts growing at spheroid perimeters but not within them (Figure S2 in File S1). MCF-7^M^ were grown at two seeding compositions, one with a fibroblast:cancer cell ratios of 1∶5 (indicating a ‘low density’ of fibroblasts) and one with a ratio of 1∶3 (a ‘high density’ of fibroblasts). In the MCF-7^M^ cultures, SNAIL and SLUG were expressed at much higher levels and E-cadherin levels were decreased 2-fold or more compared to MCF-7^S^ cultures (Fig. 2*B*). A density-dependent effect on expression level of these genes was observed between the low and high fibroblast ratios. Because the mRNA levels shown represent a mixture of the cancer cells and fibroblasts, mRNA levels for these markers from HMF grown alone are also displayed. Basal levels of SNAIL and SLUG in HMF are higher than found in MCF-7 cells, but this is only approximately 30% the level observed in the co-culture, suggesting that regulation of the EMT phenotype is synergistic and not dominated by one cell type. Reduction in E-cadherin at the mRNA level was also confirmed at the protein level by immunohistochemistry (Fig. 2*C*).

To demonstrate that the presence of HMF also altered the behavior of cancer cells, MCF-7 grown in a Transwell co-culture with HMF were found to be significantly (p = 0.0045) more invasive compared to MCF-7 alone (Fig. 2*D*). Comparing the mixed and sandwich culture models, we observed that increasing both the co-culture time and the relative number of fibroblasts in the co-culture induced similar trends in the expression of EMT markers in the cancer cells. These results suggest that fibroblast interactions play a significant role in altering the ER^+^ cancer cell phenotype.

### Co-culture with mammary fibroblasts down-regulates ERα in MCF-7 and drives hormone-independent proliferation

**Figure 3:**
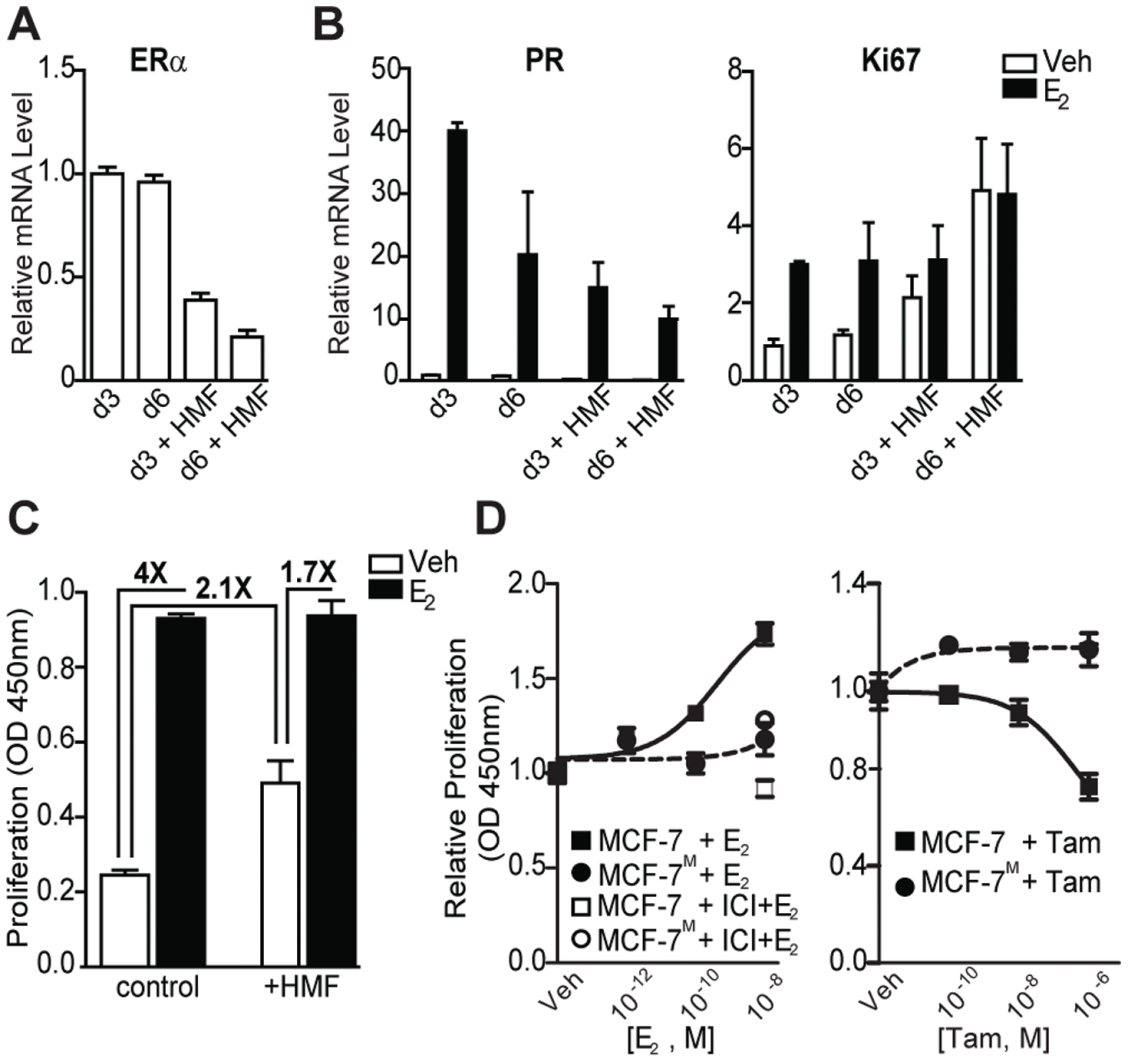
Co-culture with human mammary fibroblasts down-regulates ERα and fosters hormone-independent growth of breast cancer cells. (A) Fibroblasts down-regulate ERα mRNA levels at 3 and 6 days and this effect increases with longer co-culture time. (B) HMF reduce the response of MCF-7 cells to estradiol, as measured by PR, and HMF increases basal Ki67 mRNA at 3 and 6 days and eliminates further increase by E_2_. (C) Co-culture with HMF increases the basal proliferation rate of MCF-7, and decreases the fold change of E_2_ stimulation of proliferation monitored at 3 days. (D) MCF-7^M^ proliferation fails to respond to increasing concentrations of E_2_ (left panel) and MCF-7^M^ growth is not inhibited by treatment with tamoxifen (right). Response in MCF-7 cells alone is shown for comparison.

We next examined whether co-culture with fibroblasts might alter response of ER^+^ cancer cells to hormones. As a prelude to using our models for this effort, the response of MCF-7 to estrogen was confirmed to be similar in monolayer and in 3D culture (Figure S3 in File S1). Interestingly, after co-culture, a sustained reduction in the mRNA level of ERα in MCF-7^S^ was observed (Fig. 3*A*). Though reduced, the remaining ERα in MCF-7^S^ cells was functional, as shown by induction of the estradiol (E_2_)-responsive genes progesterone receptor (PR) and Ki67 mRNA after treatment (Fig. 3*B*). The basal levels of Ki67 mRNA and cell proliferation were increased in the co-cultures, and after 6 days, the response to E_2_ was greatly diminished, indicating that MCF-7^S^/MCF-7^M^ proliferation became increasingly hormone-independent (Fig. 3*B and 3C*). Accompanying this hormone-independent growth, the antiestrogen tamoxifen (Tam) was no longer effective in inhibiting the growth of MCF-7^M^ (Fig. 3*D*).

### Conditioned medium from 3D co-cultures induces EMT in breast cancer cells and also in normal mammary epithelial cells

**Figure 4:**
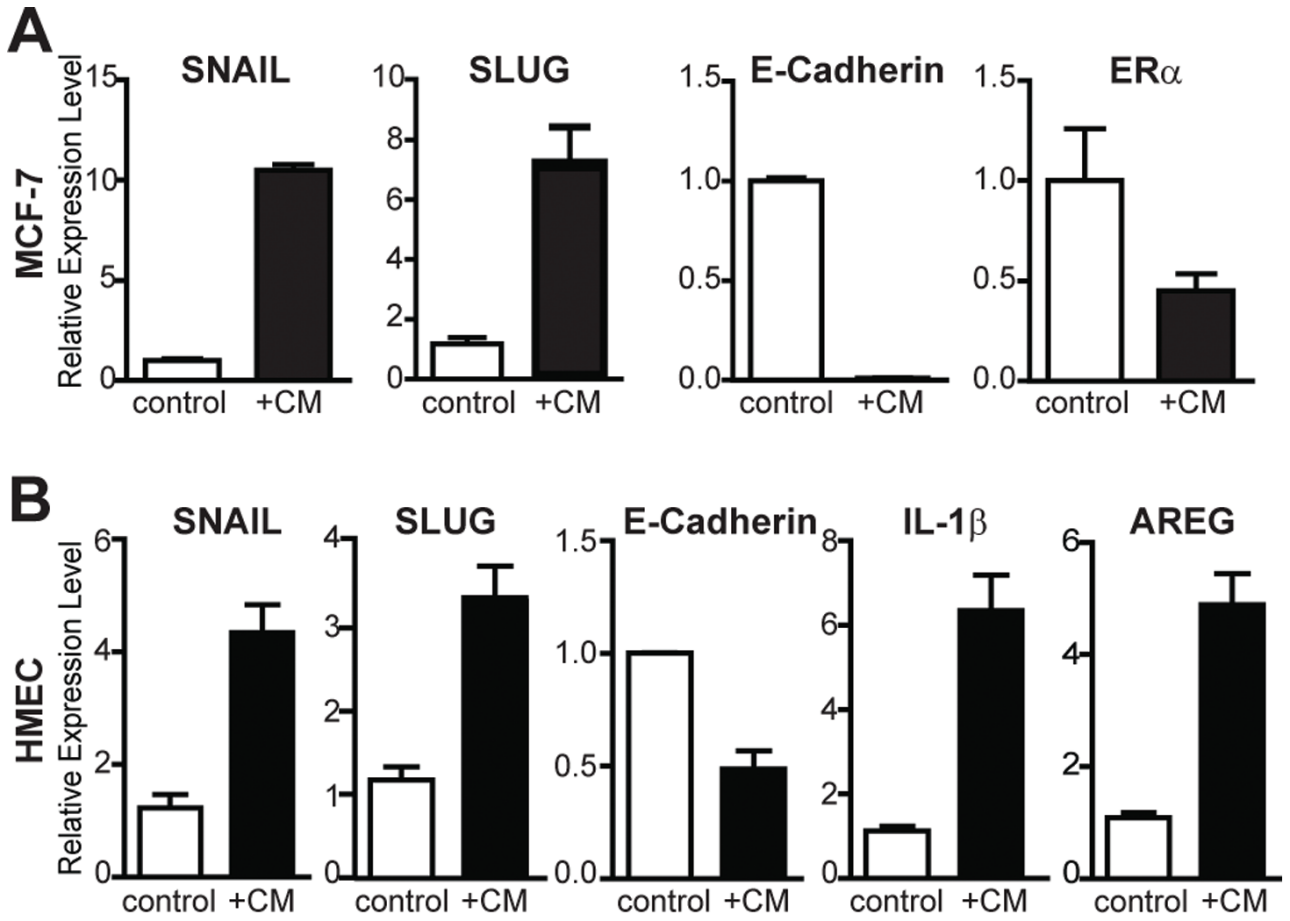
Treatment of cancerous and normal breast cells with conditioned medium (CM) alters gene expression. (A) Gene expression changes in MCF-7 cells treated with CM reveal a 10-fold increase in SNAIL, a 7-fold increase in SLUG, and elimination of detectable E-Cadherin mRNA. The increase in EMT markers is accompanied by a down-regulation of ERα mRNA. (B) After treatment of HMEC grown in monolayer culture with CM, there were changes in expression of EMT markers and increases in IL-1β and the growth factor amphiregulin (AREG), suggesting an important role for paracrine signaling between the tumor microenvironment and adjacent normal epithelium.

To investigate a possible role for paracrine signaling and soluble factors in driving alterations in breast cancer cells, we examined gene expression changes in MCF-7 cells treated with conditioned medium (CM) (Fig. 4*A*). Notably, ERα levels were halved in CM-treated samples, and EMT markers SNAIL and SLUG were upregulated with an almost complete loss of E-Cadherin mRNA (Fig. 4*A*). To examine whether the cancer cell-fibroblast CM might also affect normal mammary epithelium, we grew normal primary human mammary epithelial cells (HMEC) with CM and monitored changes in gene expression. Strikingly, the same EMT phenotype was observed although less dramatically than in MCF-7 cells (Fig. 4*B*). Based on these results, we propose that fibroblast-cancer cell interactions can induce an EMT in nearby normal breast epithelial as well as cancer cells via soluble protein signaling. These observations imply that the environment generated by fibroblast-cancer cell interactions may promote aggressive cell phenotypes in both normal and cancerous epithelial cells.

After establishing that fibroblast-cancer cell interactions occur via paracrine signaling, we analyzed the CM with a protein array in order to elucidate the molecular components involved in this interaction. Of the 507 proteins measured on the array, 46 were found to be upregulated five-fold or greater in the co-culture CM compared to naïve MCF-7 CM (Table 1). Notably, many of the upregulated proteins were members of the matrix metalloproteinase family (MMPs), which has been previously implicated in aggressiveness of breast cancer cells [28] and human tumors [29]. Other identified proteins included growth factors and cytokines that play a role in recruiting cells involved in the inflammatory response. Functional classification of the secreted proteins also showed enrichment in components of chemotaxis and ECM remodeling.

**Table 1:** Secreted proteins that were increased 5-fold or higher in co-culture conditioned medium (CM) compared to CM from naïve MCF-7.

CCL14/HCC-1/HCC-3	MUSK
EDA-A2	NAP-2
ENA-78	NOV/CCN3
Endoglin/CD105	Orexin B
FGF-8	Osteoactivin/GPNMB
GCP-2/CXCL6	PD-ECGF
GRO	PDGF R α
IGF-I	PDGF R β
IGF-I SR	PDGF- BB
IL-1β	RELM β
IL-2 R γ	SDF-1/CXCL12
Lck	Sgp130
Latent TGF-β bp1	TGF-β RII
Lymphotoxin β R / TNFRSF3	Thrombospondin-4
M-CSF R	Thymopoietin
MFG-E8	TIMP-3
MIP 2	TIMP-4
MMP-1	TRAIL R1/DR4/TNFRSF10A
MMP-3	Trail R3/TNFRSF10C
MMP-13	VEGF R2 (KDR)
MMP-14	VEGF-D
MMP-19	WIF-1
MMP-25/MT6-MMP	XEDAR

### Patients with high expression of the secreted protein signature are more likely to have poor clinical outcomes

**Figure 5:**
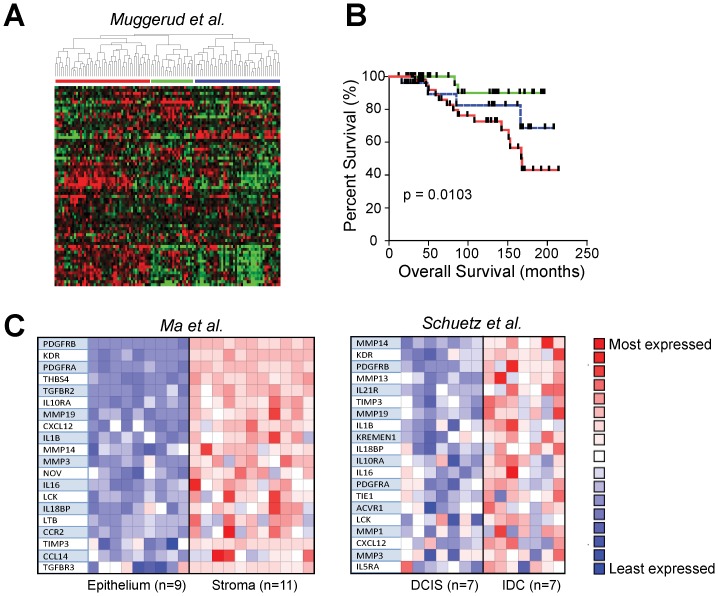
Hierarchical clustering of breast cancer patient samples based on the secreted protein signature reveals distinct subgroups. (A) Hierarchical clustering of primary tumors reveals three distinct classes (B) Kaplan–Meier method and the log-rank test was used to compare the mean survival rates across the three identified classes. Patients in the red class (i.e. with higher gene expression of the signature secreted proteins) are significantly associated with a poorer prognosis. (C) ONCOMINE analysis shows that the protein signature is also overexpressed in IDC compared with DCIS in an independent dataset. The protein signature is also significantly correlated with the stroma of breast tumors

To examine whether our *in vitro* secreted protein signature could be used to complement currently-available approaches for stratification of breast cancer patient samples, we utilized a publicly available dataset [30] comprised of 109 patients diagnosed with Invasive Breast Cancer (IDC), pure Ductal Carcinoma *in Situ* (DCIS), or a mixed diagnosis (IDC with a DCIS component). Genes associated with upregulated CM proteins (iSig) were used to cluster microarray data from these primary tumors. Three primary clusters were found (Fig. 5*A*). The red cluster, characterized by elevated levels of MMPs and TGFβ, showed a significantly worse prognosis (Fig. 5*B*). Then, ONCOMINE analysis was used to validate results across additional breast cancer datasets [31]–[34]. The iSig was strongly correlated with invasion and poor prognosis, and was associated with stromal gene profiles in microdissected samples (Fig. 5*C* and Figures S5 and S6 in File S1). The utility of a signature derived from cell culture models to stratify patient data based on gene expression suggests that, after further work and validation, bottom-up protein signatures may be useful to monitor the dynamic states of tumor progression. To our knowledge, this is the first study that utilizes a secreted protein signature to stratify breast cancer patient outcome.

### FT-IR spectroscopic imaging can be used to monitor hormonal response of cancer cells

**Figure 6:**
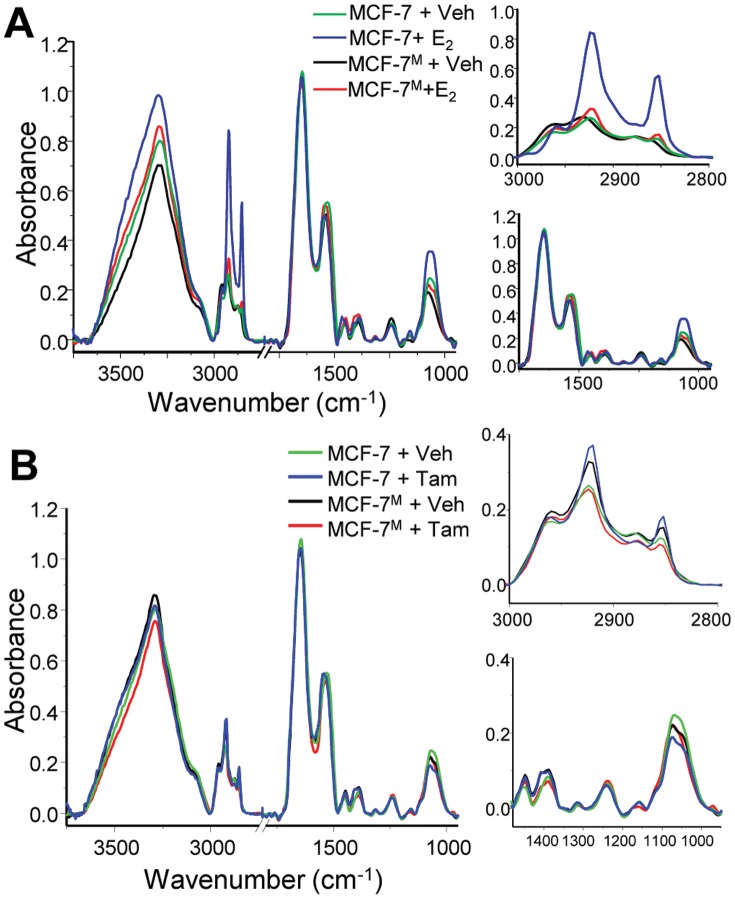
Fourier transform-infrared (FT-IR) spectroscopic imaging can be used to monitor hormone response in cells in culture. (A) A change in FT-IR spectroscopic imaging is seen in MCF-7 cells treated with estradiol (E_2_), particularly in the C-H stretching region (3000 – 2750 cm^−1^) and in the peak associated with nucleic acids (1080 cm^−1^). However, when cells are co-cultured with human mammary fibroblasts (HMF), the response to hormone is lost. (B) Spectral changes are also seen in MCF-7 cells treated with tamoxifen (Tam). While there is a slight induction in peaks associated with the C-H stretching region upon treatment with Tam, the peak associated with nucleic acids, and therefore proliferation, is decreased in MCF-7 cells. This is correlated with the anti-proliferative effects of tamoxifen on ER^+^ cells. In samples that have been co-cultured with HMF, this change is not seen at the 1080 cm^−1^ peak, corresponding to the endocrine-resistant growth that was seen using proliferation assays.

Our data show that ER level and activity can be dynamically altered and that ER-positive cells may not respond to endocrine therapy if they have undergone EMT. Given the spatially-heterogeneous nature of tumors, an imaging technique to record the individual cellular states within a tumor is needed. While IHC can provide information about protein expression, it is time-consuming and requires antibodies [35]. We hypothesized that using FT-IR microscopy could be used to measure the chemical response of breast cancer cells to hormone treatment or to endocrine therapy without loss of spatial information. We used the same 3D culture samples and conditions so that mRNA expression and FT-IR signatures could be directly compared. After E_2_ treatment, we observed a large increase in the C-H vibrational region of the spectrum (Fig. 6*A*), commonly associated with the CH_2_ bending of fatty acids and lipids [36]. These three peaks (2960 cm^−1^, 2930 cm^−1^, and 2850 cm^−1^) are also correlated with fatty acyl chain peroxidation^43^. Similarly, the peak at 1080 cm^−1^, associated with the phosphate backbone of nucleic acids and altered metabolic activity, is increased upon E_2_ treatment. Thus, changes in absorption pattern can be correlated with biological changes occurring in ER^+^ breast cancer cells in response to treatment with E_2_.

Next we sought to differentiate between cells having functionally active ER and those that do not. Samples from the mixed co-culture were imaged, and the average spectra upon treatment with E_2_ are shown (Fig. 6*A*). Analysis of the spectra revealed that there was a loss of the chemical signature associated with E_2_ response in the co-cultured samples, corresponding to the reduction of ERα and ERα-mediated response in MCF-7^M^. The fibroblast dose-response seen in mRNA levels was also apparent in IR spectra (Figure S7 in File S1). The loss of E_2_ response was observed in the IR spectra of CM-treated samples, but to a lesser extent than in the direct co-culture (Figure S8 in File S1). The chemical signature of E_2_ treatment and its loss after co-culture with HMF in 3D culture reveals that FT-IR imaging may be useful in determining ER function in cells *in vitro*.

The cell response to tamoxifen also had a distinct associated spectral signature (Figure 6*B*). There was a change in the peaks located in the C-H stretching region of the spectrum in MCF-7 after treatment with Tam. Additionally, the anti-proliferative effects of Tam were specifically seen in reductions in absorbance at the 1080 cm^−1^ peak. Similarly, the loss of response to Tam in the MCF-7^M^ co-culture is correlated specifically to a spectral signature. While the 1080 cm^−1^ peak was substantially decreased in MCF-7 after Tam treatment, the peak was unchanged in MCF-7^M^. Thus, this label-free approach can discriminate between cells that are responsive and those that are resistant to tamoxifen treatment.

**Figure 7:**
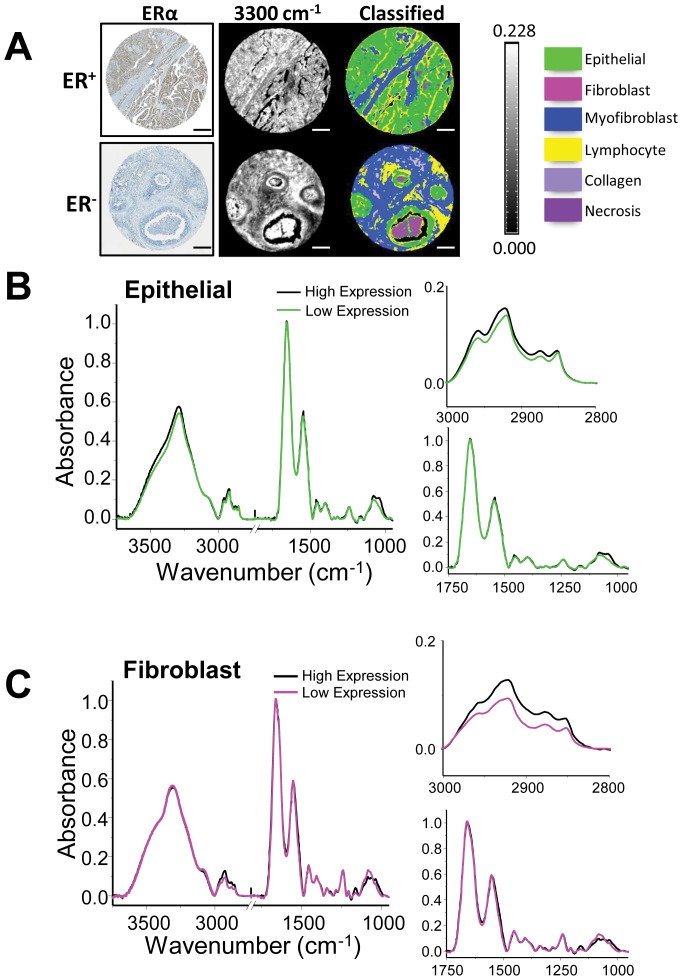
Spectroscopic signatures determined using 3D co-culture models can be translated to human breast tissue samples. (A) Tissue microarray (TMA) biopsy cores (1.5 mm core diameter) were IHC stained for ERα and also imaged using FT-IR imaging (N-H/O-H band at 3300 cm^−1^ visualized here for clarity). Images classified using Bayesian classifier are displayed as well to highlight the ability of FT-IR to discriminate between cell types in complex samples. Scale bar represents 250 µm. (B) Differences between epithelial pixels in patient samples with high (>80%) and low (<20%) expression of ERα can be seen in peaks at 1080 cm^−1^ (phosphate) and 1030 cm^−1^ (glycosidic bonds). Interestingly, there are more apparent differences in these peaks when pixels from fibroblasts are analyzed. Full spectrum (3800 – 950 cm^−1^), C-H stretching region (3000 – 2750 cm^−1^), and biochemical fingerprint region (1750 – 950 cm^−1^) are shown.

Next, unstained human breast tumor tissue microarrays (TMAs) containing samples from 148 patients were imaged with FT-IR in order to establish proof of principle on whether spectral differences in ER status would be apparent in clinical samples. ER expression was determined by IHC staining for ERα on an adjacent TMA section. Spectra of epithelium from IDC samples with high expression of ER (>80% of cells ER^+^) were extracted and compared to IDC samples with low expression of ER (<20% of cells ER^+^) (Fig 7). A Bayesian-type classifier was developed for the histologic discrimination of breast tissue (Fig 7*A*) using previously reported methods [18]. The classifier was used to extract pixels corresponding to epithelium or fibroblast regions from breast cancer samples with varying levels of ER. Spectroscopic signatures of different cell types, namely epithelial/cancer cells and fibroblasts, were compared. Several spectral markers could be translated from these 3D culture models to human tissue. In the epithelium (Fig 7*B*), spectra from samples with high ER expression demonstrated similar absorbance in the C-H stretching region (3000 – 2825 cm^−1^) to MCF-7 3D spheroid cultures, and the overall absorbance in this region was higher than samples with low ER expression. Strikingly, significant spectral differences were observed in fibroblasts from tumors with different ER levels (Fig 7*C*). These results suggest that IR might be used to identify features in the stromal compartment that are associated with endocrine response. Spectroscopic differences between human breast tumor samples with varying levels of ER expression suggest that FT-IR imaging can complement IHC analysis by providing additional chemical information about the function of this nuclear hormone receptor in breast tumors.

## Discussion

Our findings reveal that the interaction of ER^+^ breast cancer cells with mammary fibroblasts promotes a more aggressive and endocrine therapy-resistant state. Mammary fibroblasts induce a downregulation in the level of cellular ERα and promote a more rapidly proliferating hormone-independent state in ER^+^ breast cancer cells. Importantly, the cross-over to hormone-independent growth is associated with acquisition of an EMT phenotype, which has been previously correlated to invasive disease and poor prognosis [37]–[40], [25]. EMT was concurrent with the disruption of E_2_/ERα signaling and the occurrence of a more invasive phenotype. We also found ERα to be downregulated such that it was no longer the prime determinant of tumor growth, underscoring this as a potential mechanism by which tumors become resistant to endocrine therapy. Of interest, a recent report has shown a similar association between low ERα and EMT and poor prognosis in human uterine endometrial carcinoma [40]. The therapeutic implication is that although women may be diagnosed as ER^+^, some will not respond favorably to endocrine therapies because growth of their tumor is regulated by the nature of their tumor microenvironment. Therefore, it may be advantageous to simultaneously inhibit paracrine factors produced by a fibroblast-enriched tumor microenvironment in combination with the use of anti-estrogen or other endocrine therapies.

In analyzing fibroblast-cancer cell interactions for molecular targets using protein microarrays, we found this interaction to result in the expression of a set of regulatory proteins, including M-CSF, SDF-1, and MMP family proteins that have been individually associated with cancer activated fibroblasts and invasive disease [28], [41], [42], [29]. Our time-course experiments show that these represent early signaling events in the fibroblast-cancer cell relationship and suggest that it is the combined external signaling of both fibroblasts and cancer cells that contributes to the extracellular milieu that drives this EMT phenotype. Notably, while the presence of fibroblasts increased tumor cell invasiveness, the proteins secreted into conditioned medium after co-culture also greatly affected the properties of normal mammary epithelial cells. These observations imply that fibroblast-cancer cell interactions may put at risk nearby normal mammary epithelial cells, initiating early cancer-like phenotypic changes that may be relevant to acceleration of early stages of breast tumorigenesis. Our observations are supported by work in mouse mammary gland showing the impact of mammary stromal cells in changing motility and promoting the initiation of cancer-like properties of normal mammary epithelial cells [42]. A recent report shows that HGF signaling from normal mammary fibroblasts is able to promote basal-like breast cancer phenotypes in a 3D culture model [43], [44]. Further, fibroblasts may be implicated in the etiology of diseases such as benign prostatic hyperplasia that have been associated with EMT [45]. While many studies have shown that cancer-activated fibroblasts (CAFs) alter tumor behavior [46]–[48], our model system captures early stages of tumor-stroma interactions. The hallmark of CAFs is stable expression of α-SMA, and there is some but very mild staining in the MCF-7^M^ cultures (Figure S9 in File S1), suggesting that the fibroblasts used in this study are also being altered by the cancer cells.

Because the secreted proteins identified in these experiments are potentially clinically actionable factors, we used the protein expression data to obtain a corresponding panel of genes (iSig). This *in vitro* signature could be used to cluster patient samples and predict prognosis. Our approach is complementary to existing subtyping based on genetic or histologic means and can be employed to broaden and enhance the present system. Whereas several signatures of breast cancer invasiveness and poor prognosis have been reported [49], [50], these are derived from a top-down approach utilizing statistical patterns found in the gene expression profiles of patient tumors. By taking the results of controlled *in vitro* experiments and comparing the data to patient genome-wide expression, our complementary ‘bottom up’ approach can be used to provide mechanistic insight into cell-cell interactions promoting cancer progression. In this way, genetic information from established tumors found in patients is related back to specific changes seen under controlled experimental settings. This allows for the determination of gene and protein features that may be seen during early stages of tumor cell migration into the surrounding stromal microenvironment.

Finally, we utilized FT-IR spectroscopic imaging to determine hormone sensitivity in biological samples. This label-free and non-destructive technique was used to identify chemical signatures of disease states. We correlated gene expression and a loss of E_2_/ERα signaling with decreases in peak height in the C-H stretching region of the spectrum in 3D culture cell samples, consistent with prior observations that EMT is associated with changes in lipid profiles in epithelial cells [51]. We also confirmed that MCF-7 cells become resistant to tamoxifen using spectral features, primarily in the peaks associated with nucleic acids. These results directly correlate optical profiles to cellular behaviors and genomics. Translating the cell culture results to patient samples, the *in vitro* chemical signature was also found in invasive breast cancer biopsies with differing levels of ER expression. These results indicate that FT-IR imaging can potentially be useful, alongside IHC and molecular marker analyses, to determine ERα functionality in patient tumor specimens. Though other imaging techniques such as positron emission tomography (PET) have been used for functional imaging studies before and after therapy [52], development of a similar approach at the microscopic scale is complicated by the need to also appreciate the cell type, morphology, and spatial phenotypic heterogeneity to understand the tumor and its microenvironment. FT-IR imaging can provide microscopic evidence rapidly and is applicable at the time of usual post-biopsy diagnoses in pathology.

In conclusion, by combining molecular profiling with chemical imaging, we have demonstrated that mammary tissue fibroblasts can alter therapeutic response to anticancer agents and play a crucial role in controlling whether ER^+^ breast cancer cells are able to respond well to hormone or become resistant to endocrine therapies. Thus, interactions with the tumor microenvironment can result in deregulation of estrogen receptor signaling and alterations in cell survival signaling molecules that provide tumors with alternative proliferative and survival stimuli [53]–[55]. It was recently shown that histologically normal fibroblasts present in negative tumor margins can promote invasive phenotypes in cancer cells [56], which supports our conclusions that the interaction between fibroblasts and cancer cells alters tumor cell behavior and therapeutic response. The pro-inflammatory microenvironment that is generated as a result of epithelial-fibroblast interactions is sufficient to stimulate EMT in breast cancer cells and, also strikingly, in normal mammary epithelium. Notably, the molecules identified from this interaction are also upregulated in breast cancers from patients with invasive disease and predict a poor clinical outcome [28], [26], [57], [27], [58], [29], [59], [25]. While the study described here was restricted to ER^+^ tumors, we hope that this model will be extended to other molecular sub-classes of breast tumors and eventually may be used to direct individualized therapies based on inclusion of the characteristics of a patient's tumor microenvironment. Our findings provide new molecular insights, coupled with changes in chemical imaging by FT-IR, into the microenvironment regulation of tumor behavior via interaction between cancer cells, fibroblasts, and nearby normal epithelial cells. These findings should aid in the development of specific inhibitors of molecular species in this crosstalk that promote growth and progression to a more aggressive, therapy-resistant state. Further, we have shown the utility in combining chemical and molecular imaging approaches in order to aid in cancer patient management by providing additional prognostic information at the time of the initial biopsy.

## Materials and Methods

### Cell Culture

MCF-7 cells were grown as previously described [27]. Primary normal human mammary fibroblasts (HMF) were purchased from ScienCell and cultured according to their specifications (ScienCell Research Laboratories, Carlsbad, California, USA). Primary normal human mammary epithelial cells (HMEC) were also purchased from ScienCell and cultured according to their specifications. HMEC were used prior to passage 3 to ensure phenotype stability. For sandwich (non-contact) co-culture, MCF-7 cells were cultured in hormone-depleted medium (TM, comprised of phenol red-free basal MEM containing 5% charcoal-dextran stripped calf serum). 3D cultures were prepared by spreading a thin layer of growth factor-reduced phenol red-free Matrigel (BD Biosciences, San Jose, California, USA) in multi-well plates and seeding a low density of MCF-7 on top (for a 12-well plate, 100 µl of Matrigel was used and 8,000 cells were seeded per well). The cells were seeded in 1 ml of TM containing 2.5% dissolved Matrigel. Fibroblasts were embedded in 600 µl of a 2 mg/ml type I collagen gel (Type I collagen from rat tail tendon, BD Biosciences, San Jose, California, USA) at a density of 350,000 cells/ml and prepared in 24-well plates as previously described [16]. MCF-7 cells were grown in 3D for 5-7 days in TM before adding fibroblast layer. Fibroblasts were grown for two days in 3D gels before co-culture. Co-culture ranged from 3-6 days before analysis. For the ‘direct’ co-culture, 100 µl of Matrigel was spread in each well of a 12-well plate and 8,000 MCF-7 cells were seeded as before. After 5–7 days of growth in TM, HMF were seeded at a ratio of 3∶1 or 5∶1 (2,700 or 1,600 HMF per well respectively). The medium was then switched to a 3∶1 or 5∶1 ratio of TM: complete FM medium (ScienCell Research Laboratories) for the duration of the co-culture. MCF-7 cells were grown alone as a control, and their medium was the 1∶1 mix for these experiments.

### Proliferation Assays

All proliferation assays were performed using the WST-1 reagent (Roche Applied Science, Indianapolis, Indiana, USA) as described [60]. Absorbance was read at 450 nm on a PerkinElmer Victor X Multilabel Plate Reader (PerkinElmer, Waltham, Massachusetts, USA). Data were normalized to the vehicle control based on cell type (for example, MCF-7 treatments were normalized to MCF-7 vehicle control, while 3∶1 treatments were normalized to the 3∶1 vehicle control) in Excel and plotted in GraphPad. All samples were prepared in duplicate.

### Invasion assays

HMF (1×10^4^) were seeded on the bottom of modified Boyden chambers while MCF-7 (3×10^4^) were seeded on precoated filters (8 µm pore size) after membrane rehydration (BD Biosciences). Following incubation for 48 h at 37°C, cells were fixed in 10% formalin buffer and stained using crystal violet. Non-invaded cells on the surface of the filter were removed using a cotton swab. Invasion was quantified as described [60] by counting the number of cells that invaded the filter compared to the total seeded number. Data are expressed as mean ± SD and P<0.05 was assigned as statistically significant by using unpaired one-tailed t tests (GraphPad Software, San Diego, CA).

### Quantitative Real-time PCR and Primers

Total RNA was isolated from cells using TRIzol (Invitrogen, Life Technologies, Carlsbad, California, USA). cDNA was prepared by reverse transcription with M-MuLV Reverse Transcriptase (New England Biolabs Inc, Ipswich, Massachusetts, USA). mRNA expression was measured using quantitative real-time PCR on a high-throughput ABI Prism 7900HT real-time instrument (Applied Biosystems, Life Technologies, Carlsbad, California, USA) using SYBR Green PCR Master Mix (Applied Biosystems) as described previously [61].

### Immunohistochemistry

For both immunohistochemistry and FT-IR imaging analysis, samples were fixed in freshly-prepared 4% paraformaldehyde for 1 h, mounted in Histogel (Thermo Fisher Scientific Inc., Waltham, Massachusetts, USA), and then paraffin-embedded. For paraffin embedding, samples mounted in Histogel were dehydrated with serial ethanol washes (50%, 70%, 80%, 95%, 100%, 100% for 3 h each) followed by three 2 h clearing steps in xylenes, and finally four 1 h paraffin infiltration steps (ParaPlast Plus, Sigma-Aldrich, St. Louis, Missouri, USA).

For IHC, samples were sectioned at 10 µm onto poly-L-lysine coated slides (Thermo Fisher Scientific, Waltham, Massachusetts, USA). Once dry, slides were deparaffinized (2×3 min xylene, 1×3 min 1∶1 xylene/100% ethanol, 2×3 min 100% ethanol, 1×3 min 95% ethanol, 1×3 min 70% ethanol, 1×3 min 50% ethanol, cold tap water). Antigen retrieval was performed by boiling the samples in the microwave for 15 min in 10 mM sodium citrate buffer (pH 6.0). Slides were blocked in Tris-buffered saline (TBS) containing 10% FBS overnight at 4°C. Primary antibody was added in TBS containing 1% BSA for one hour at room temperature. The remainder of the protocol was performed as specified using a HRP/DAB detection kit (EXPOSE Mouse and Rabbit Specific HRP/DAB Detection IHC kit, Abcam, Cambridge, Massachusetts, USA). E-cadherin antibody was used at a dilution of 1∶400 (E-cadherin 24E10 Rabbit mAb, Cell Signaling Technology, Danvers, Massachusetts, USA). α-SMA antibody was used at a dilution of 1∶200 (α-SMA clone 1A4, Dako Denmark A/S, Glostrup, Denmark).

### Antibody Arrays and Classification of the Protein Signature

Antibody Array was from RayBiotech, Inc. (L-Series 507: RayBio Label-based Human Antibody Array 1-Membrane). Array analyzes 507 human proteins simultaneously in two samples to examine differential expression. Conditioned medium was obtained from mixed (direct contact) co-cultures after 3 days and this was compared with conditioned medium from MCF-7 cells grown in 3D for the same length of time. Samples were prepared according to the manufacturer's specifications. Protein array membrane was imaged with an Image Quant LAS 4010 Luminescent image analyzer (GE Healthcare, Waukesha, Wisconsin, USA). GO and pathway classifications of the protein signature were conducted using web-based DAVID Bioinformatics Resources database (Figure S4 in File S1) [62] and MetaCore (Thomson Reuters, New York, NY, USA).

### FT-IR Imaging and Image Classification

For cell culture samples, paraffin embedded samples were sectioned at 5 µm onto MirrIR IR-reflective glass slides (Kevley Technologies, Chesterland, Ohio, USA). Once dry, samples were deparaffinized in hexanes for 24 h and then dried before imaging. All tissue was commercially-obtained (BR961 and BR1003 Breast Tissue Microarrays, US Biomax, Inc., Rockville, Maryland, USA). Unstained sections were placed on barium fluoride substrates (International Crystal Laboratories, Garfield, New Jersey, USA), deparaffinized in hexanes for 24 hours, and completely dried before imaging. Adjacent sections of the arrays were stained for morphology (Hematoxylin and Eosin) and for ERα expression (IHC). For FT-IR imaging, a PerkinElmer Spotlight 400 (PerkinElmer, Waltham, Massachusetts, USA) equipped with a thermal source and a raster-scanning linear array detector was used with a Germanium ATR imaging accessory. An NB medium apodization was applied, a 1 cm s^−1^ mirror speed was used for acquisition, and zeropadding was not used. Background scans were taken at 8 cm^−1^ spectral resolution. 150×150 µm images were collected at 8 cm^−1^ spectral resolution with 16 scans per pixel and a 1.56×1.56 µm pixel size. Data were atmospheric- and ATR-corrected on the Spotlight, and further processing was done using ENVI-IDL. A minimum noise fraction (MNF) algorithm was applied on all images in ENVI-IDL [63] to reduce noise in the data. Two regions were imaged per sample, and samples were prepared in duplicate (for a total of 4 data sets per condition). Because each image represented a combination of cells, Matrigel, and Histogel, a Bayesian-type classifier was developed as previously described [18] that labeled spectral contributions from cells within the image. Briefly, 10–15 images of ‘pure Matrigel’, ‘pure Histogel’, and samples containing Histogel, Matrigel, and cells were imaged. Several thousand pixels were identified and manually labeled as coming from one of three classes: Matrigel, Histogel, and cells. A three-class Bayesian-type classifier was developed using this training set and was subsequently validated on an independent set of samples. This allowed us to extract pixels from the image that corresponded to cells rather than extracellular matrix or embedding compounds. All spectra displayed here represent the average spectra from the epithelial cells present in the sample. Once spectra were classified and extracted, a baseline correction was applied and spectra were further normalized to the Amide I peak (1656 cm^−1^) for comparison. Spectra were plotted using OriginPro software. Similar approaches to FT-IR imaging and classification have been described in the literature [22], [18], [64], [65], [20], [66]–[68].

### Data Analysis of Microarrays

A publicly-available microarray dataset was downloaded from GEO (GSE26304) and included 31 pure DCIS patients, 36 IDC patients, and 42 patients with ‘Mixed’ histology [30]. Data were transformed into GeneSpring GX 7.3 and chips and genes were median normalized and median polished. Hierarchical clustering of the genes matching the 46-protein secreted signature was performed and displayed using Eisen Cluster and TreeView software for analysis and visualization. Kaplan–Meier method and the log-rank test was used to compare the mean survival rates across the groups identified by hierarchical clustering.The 46-protein signature was uploaded in ONCOMINE (Compendia Bioscience, Ann Arbor, MI), using the following filters: odds ratio >2, and P-value <0.001, epithelial versus stroma or invasive cancer versus ductal-carcinoma *in situ* and cancer type (breast cancer). The Oncomine database was also used for visualization of significantly associated datasets.

## Supporting Information

File S1 **Figures S1-S9.** Figure S1. Amphiregulin mRNA expression is increased in MCF-7 breast cancer cells in the sandwich co-culture (MCF-7^S^). Figure S2. Light microscopy image showing fibroblasts recruited to MCF-7 spheroids after 3 and 5 days in culture. Figure S3. Comparison of morphology and response to E_2_ in 2D and 3D MCF-7 models. (A) Micrograph of MCF-7 cells grown in 2D and 3D culture. (B) Response of MCF-7 cells to E_2_ stimulation is similar in 2D and 3D cultures as monitored by induction of E_2_ response genes progesterone receptor (PR) and Ki67. Figure S4. Table of DAVID pathway association based on iSig. Figure S5. Table of ONCOMINE dataset patient characteristics. Figure S6. Expression of iSig is correlated with breast cancer stromal expression. Figure S7. FT-IR signature of E_2_ response of MCF-7 cells is based on fibroblast density. Figure S8. FT-IR signature of E_2_ response of MCF-7 cells is affected by treatment with CM. Figure S9. Fibroblasts recruited to the outside of MCF-7 spheroids display mild α-SMA staining.
(PPTX)
